# PRRX1-TOP2A interaction is a malignancy-promoting factor in human malignant peripheral nerve sheath tumours

**DOI:** 10.1038/s41416-024-02632-8

**Published:** 2024-03-06

**Authors:** Shota Takihira, Daisuke Yamada, Tatsunori Osone, Tomoka Takao, Masakiyo Sakaguchi, Michiyuki Hakozaki, Takuto Itano, Eiji Nakata, Tomohiro Fujiwara, Toshiyuki Kunisada, Toshifumi Ozaki, Takeshi Takarada

**Affiliations:** 1 Department of Regenerative Science, Okayama University Graduate School of Medicine, Dentistry and Pharmaceutical Sciences, Okayama, 700-8558 Japan; 2 Department of Orthopedic Surgery, Okayama University Graduate School of Medicine, Dentistry and Pharmaceutical Sciences, Okayama, 700-8558 Japan; 3 Department of Cell Biology, Okayama University Graduate School of Medicine, Dentistry and Pharmaceutical Sciences, Okayama, 700-8558 Japan; 4 Department of Orthopedic Surgery, Fukushima Medical University School of Medicine, Fukushima, 960-1295 Japan

**Keywords:** Sarcoma, Protein-protein interaction networks

## Abstract

**Background:**

Paired related-homeobox 1 (PRRX1) is a transcription factor in the regulation of developmental morphogenetic processes. There is growing evidence that *PRRX1* is highly expressed in certain cancers and is critically involved in human survival prognosis. However, the molecular mechanism of PRRX1 in cancer malignancy remains to be elucidated.

**Methods:**

*PRRX1* expression in human Malignant peripheral nerve sheath tumours (MPNSTs) samples was detected immunohistochemically to evaluate survival prognosis. MPNST models with *PRRX1* gene knockdown or overexpression were constructed in vitro and the phenotype of MPNST cells was evaluated. Bioinformatics analysis combined with co-immunoprecipitation, mass spectrometry, RNA-seq and structural prediction were used to identify proteins interacting with PRRX1.

**Results:**

High expression of *PRRX1* was associated with a poor prognosis for MPNST. *PRRX1* knockdown suppressed the tumorigenic potential. *PRRX1* overexpressed in MPNSTs directly interacts with topoisomerase 2 A (TOP2A) to cooperatively promote epithelial-mesenchymal transition and increase expression of tumour malignancy-related gene sets including mTORC1, KRAS and SRC signalling pathways. Etoposide, a TOP2A inhibitor used in the treatment of MPNST, may exhibit one of its anticancer effects by inhibiting the PRRX1–TOP2A interaction.

**Conclusion:**

Targeting the PRRX1–TOP2A interaction in malignant tumours with high *PRRX1* expression might provide a novel tumour-selective therapeutic strategy.

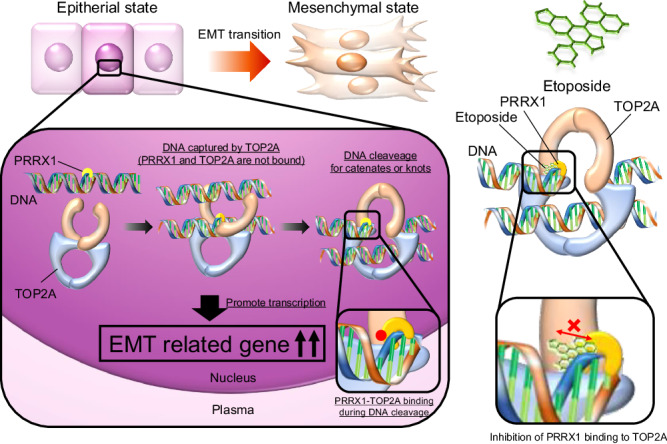

## Introduction

Paired related-homeobox 1 (PRRX1) is a member of the paired-type family of homeobox transcription factors, which have important functions in the regulation of developmental morphogenetic processes [1–3]. During mouse development, *Prrx1* is highly expressed in limb bud mesenchymal or craniofacial-mesenchymal cells and mice lacking *Prrx1* or humans with a *PRRX1* mutation die perinatally due to craniofacial and limb malformations [4–6]. Recently, Prrx1 was reported to have oncogenic or tumour-suppressive functions in several tumours. In glioma and pancreatic cancer, *Prrx1* is highly expressed in tumour-initiating cells and is involved in the regulation of invasion or metastasis [7–9]. In the sarcoma research field, mice eventually develop osteosarcoma with p53 and Rb deficiency in Prrx1-positive cells or osteoblasts, indicating a critical role for Prrx1-positive cells in osteosarcoma development [10–13]. However, the oncogenic function of PRRX1 has not been defined.

Malignant peripheral nerve sheath tumours (MPNSTs) are a subtype of soft-tissue sarcomas of neural crest and neural tube origin [14]. These tumours are rare, with an expected incidence of 0.1/100,000 per year [15, 16]. However, MPNSTs account for 5–10% of all soft-tissue sarcomas and approximately 80% of MPNSTs are pathologically indicated as high-grade malignant tumours, with a high incidence of local recurrence (21–65%) and distant metastasis (28–68%). Currently, the 5-year survival rates of MPNST patients are still only 30–50%, even with multidisciplinary treatments such as aggressive surgery, high-dose adjuvant chemotherapy and radiotherapy [14–19]. Unfortunately, clinical studies of alternative treatment strategies, such as targeted agents, have thus far shown disappointing results and have changed little [17–20]. To improve the prognosis of MPNST patients, it is clear that novel treatments are needed; thus, the identification of novel therapeutic targets is critical. It is therefore vital to fully elucidate the molecular mechanisms that support the invasion and proliferation of MPNST cells.

Here, we examined the correlation between *PRRX1* expression level and prognosis in MPNST patients. We further investigated the oncogenic role of PRRX1 using the human MPNST cell line HS-PSS.

## Materials and methods

### Patients and specimens

A total of 23 patients who underwent surgical resection for MPNST from 1998 to 2019 at Okayama University Hospital were selected as subjects for this study. The patients’ medical records, including age at diagnosis, sex, tumour size (diameter), local recurrence, metastasis status and survival time, were obtained. Written informed consent was obtained from all participants before this study and the Okayama University Hospital Institutional Review Board approved the use of all human specimens (Approval code; 1908-001). All samples and medical data used in this study were irreversibly anonymised.

### Xenograft models

The Okayama University Animal Care and Use Committee approved the experiments using mice and animal care procedures. Four-week-old female congenitally immune-deficient nonobese diabetic/severe combined immune-deficient (NOD/SCID) mice were purchased from Charles River Laboratories (Bar Harbor, MI, USA). On day 0, the mice were anaesthetised with 3% isoflurane and 100 μL containing 2.0 × 10^7^ cells of HS-PSS/Ctrl (*n* = 3), HS-PSS/shPRRX1#1, (*n* = 3), HS-PSS/shPRRX1#2, (*n* = 3) cells was injected into the both side flanks. The volumes of tumours were calculated using the formula: *L* × *W*^2^ × 0.5, where *L* is the length and *W* is the width of each tumour, as reported previously [21]. For evaluation of lung metastases, mice were sacrificed and lungs were removed and sliced (2 mm). H&E staining of the extracted lung tissue and immunostaining of PRRX1 (NBP2-13,816; Novusbio) were performed and the number of lung metastases was counted by two observers. Ki-67 (ab16667; Abcam, Cambridge, MA, USA) and terminal dUTP nick-end labelling (TUNEL) staining (Sigma-Aldrich) were also performed.

### Immunohistochemistry (IHC) and immunofluorescence (IF) analyses

Immunohistochemistry or Dab stain was performed as previously described [21]. The following antibodies were used; PRRX1-specific antibody (1:200 dilution, HPA-051084; Sigma Aldrich, USA and MA5-26579, OTI6A4; Thermo Fisher Scientific, USA), TOP2A-specific antibody (1:200 dilution; NM_001067; OriGene Technologies, Inc., Beijing, China). Three independent investigators including a pathologist evaluated and scored the sections in 10 random visual fields for each section. Using a modified Allred score as described previously, every tumour was evaluated according to the intensity of the nuclear staining (no staining = 0, weak staining = 1, moderate staining = 2, strong staining = 3) and the proportion of stained cells (0–5% = 0, 6–25% = 1, 26–50% = 2, >50% = 3). The final immunoreactive score was determined by adding together the intensity scores and the proportion of stained cells scores, yielding a minimum score of 0 and a maximum of 6. If the staining variation of intensity and proportion was observed, we employed the maximal score of staining intensity and counted all of the positive cells even with minimal intensity throughout the slide. The sum scores >3 were believed to represent significant overexpression and considered positive to simplify data analysis [21–24]. Details of the immunofluorescence have been documented in the supplementary section (Supplementary Materials & methods).

### Cells and cell culture

Three human MPNST cell lines (FMS-1, Hs-Sch-2 and HS-PSS), three human glioblastoma cell lines (U87MG, U87ΔEGFR and U251T3-mcherry), two pancreatic cancer cell lines (PANC1 and BxPC3) and one small cell lung cancer cell line (H211) were used in this study. FMS-1 was provided by Fukushima Medical University School of Medicine, Japan. Hs-Sch-2 and HS-PSS were purchased from RIKEN cell bank (Japan). U87MG, U87ΔEGFR and U251T3-mcherry were provided by department of neurological surgery, Okayama University Graduate School of Medicine, Dentistry and Pharmaceutical Sciences, Japan. PANC1 and BxPC3 were provided by Department of Cell Biology, Okayama University Graduate School of Medicine, Dentistry and Pharmaceutical Sciences, Japan. H211 were provided by Department of General Thoracic Surgery and Breast and Endocrinological Surgery, Okayama University Graduate School of Medicine, Dentistry and Pharmaceutical Sciences, Japan. LentiX293T cells were purchased from Takara Bio (Japan) and MRC-5 cells were purchased from RIKEN cell bank (Japan). Details of cell culture have been documented in the supplementary section (Supplementary Materials & methods).

### Construction of expression plasmids and transfection

To construct lentiviral vectors, the small-hairpin RNA (shRNA) was designed using the *PRRX1* and *TOP2A* Refseq cDNA sequences (GenBank accession number NM153821 and NM001067) as previously described by our group [21]. pLKO.1puro, a lentiviral vector plasmid purchased from Addgene (#8453; Watertown, MA, USA), was digested with AgeI and EcoRI and then ligated with annealed primers using Ligation High ver 2 (Takara, Japan). Primers used are listed in Table S1.

To design piggyBAC system-based eukaryotic expression vectors, the entry vectors pENTR-3xFLAG-PRRX1A (Full-length) or pDONR-3xHA-TOP2A (Full-length), were synthesised artificially using the GeneArt service (ThermoFisher, USA). piggyBAC destination vectors, PB-TAC-ERP2(#80478) or PB-TAG-ERN (#80475), were purchased from Addgene (#8453; Watertown, MA, USA) and were recombined with entry vectors using Gateway LR Clonase Enzyme MIX (ThermoFisher, USA) to construct PB-TAC-ERP2-3xFLAG-PRRX1A and PB-TAG-ERN-3xHA-TOP2A plasmids.

To design pCMViR-TSC-based eukaryotic expression vectors [25], pENTR-3xFLAG-PRRX1A (Full-length) and primers listed in Table S2 were used to prepare 3xFLAG-PRRX1A deletion fragments. The pCMViR-TSC vector was digested with BamH1 and Xho1 and then ligated with each deletion fragment using Ligation High ver 2 (Takara, Japan).

To design the pLEX307 (Addgene, USA, #41392)-based eukaryotic lentiviral expression vector, pDONR-3xHA-TOP2A (Full-length) and pLEX307 were recombined with entry vectors using Gateway LR Clonase Enzyme MIX (ThermoFisher, USA) to construct pLEX307-3xTOP2A (Full-length).

### Lentiviral production

For lentiviral production, pLKO.1 puro constructs were transfected together with packaging vectors, pMDLg/pRRE, pRSV-Rev and pMD2.G, into lentiX293T cells using PEI-MAX reagent (Polysciences, Warrington, PA, USA). Culture media were replaced with fresh media 12 h after transfection. Cells were cultured for 48 h and culture supernatants containing lentivirus were passed through a 0.45 µM PVDF filter (Hawach Scientific, Xi’an, China). Lentivirus solutions were stored at −80 °C until use. For lentiviral infection, HS-PSS cells were treated with the lentiviral solution for 24 h. After culturing for another 24 h without lentivirus, cells were treated with 1 µg/mL puromycin (Wako) to select lentivirus-infected cells.

### Establishment of HS-PSS lines using the doxycycline-inducible expression system

A NEPA21 type II electroporator (Nepagene, Chiba, Japan) was used to electroporate 1 × 10^6^ HS-PSS cells with 5 µg of the appropriate expression vector (PB-TAC-3xFLAG-PRRX1A, PB-TAC-3xFLAG-PRRX1B, PB-TAG-3xHA-TOP2A) and 5 µg of the pBase transposase vector. Cells were treated with 10 µg/mL puromycin or 500 µg/mL G418 for 3 days to select cells harbouring the piggyBAC expression system.

### Immunoprecipitation and mass spectrometry

Transfected MPNST cells (1.0 × 10^7^) and wildtype MPNST cells as a negative control (1.0 × 10^7^) were lysed with 500 μL lysis buffer containing 150 mM NaCl, 10 mM Tris-HCl (pH 7.4), 30 mM sodium pyrophosphate, 10 mM NaF, 1% NP-40, 0.5 mM EDTA and one Complete Protease Inhibitor Mixture Tablet TM (Roche, Belgium). After centrifugation at 13000 × *g* for 30 min, 60 μL supernatant was reserved as input and the rest of the extract was immunoprecipitated with anti-FLAG magnetic beads (MBL, Japan) or anti-HA beads (MBL, Japan) by gentle rotation at 4 °C for 2 h. The beads were washed three times with 10 mM PBS and 0.05% Tween-20. Precipitated proteins were eluted from the beads using SDS-PAGE sample buffer containing 150 mM NaCl, 1 mM Tris-HCl (pH 6.7), 20% sodium dodecyl sulphate, 2 M sucrose, 0.04% bromophenol blue and 1 M dithiothreitol at room temperature for 5 min. Eluted samples were boiled at 95 °C for 5 min. Samples were subjected to SDS-PAGE followed by silver staining using a Silver Stain Kit (Bio-Rad Laboratories, USA). Visible bands were then analysed by mass spectrometry (Shimadzu Techno-Research, Japan) to allow protein identification.

### RNA-sequence analysis

Total RNA was extracted using an RNeasy kit (Qiagen) and sequencing libraries were prepared using a KAPA RNA HyperPrep Kit with RiboErase (HMR) (Kapa Biosystems, USA) and a SeqCap Adapter Kit (Set A or Set B, Roche, USA) according to the manufacturer’s instructions. Sequencing libraries were transferred to an AZENTA (Suzhou, China) and were loaded onto a HiSeq 2500 system (Illumina, USA) for sequencing. All sequence reads were extracted in FASTQ format using the CASAVA 1.8.4 pipeline. Fastp (version 0.23.2) was used to remove adaptors and filter raw reads of < 60 bases in addition to leading and trailing bases with a read quality of less than 30. Filtered reads were mapped to hg19 using HISAT2 (v2.2.1). Raw counts for each gene were based on sense-strand data obtained using featureCounts software from the Subread package. RUVSeq (Release 1.28.0) was used for further normalisation to account for sample variations. Differentially expressed genes were identified through NOISeq (version 2.46.0) analysis with a threshold of prob >0.8 and abs (Log2FC) > 1. The raw and processed RNA-seq data were deposited in the NCBI GEO database under accession number GSE 214899. All normalised gene expression data were submitted to GSEA4.2.3 software or enrichR (Release 3.0) and enrichment analysis was performed. In GSEA, analyses were performed on all MSigDB gene sets and in enrichR, analyses were performed on BioPlanet, GO Biological Proccess, GO Cellular Component, GO Molecular Function, KEGG, MSigDB Hallmark and MSigDB Oncogenic Signatures.

### Protein purification and immunoprecipitation

For expressing recombinant proteins, each expression vector (pCMViR-3xFLAG-PRRX1A (Full-length), pCMViR-3xFLAG-PRRX1A (delC, 1-62), pCMViR-3xFLAG-PRRX1A (delC, 1–88), pCMViR-3xFLAG-PRRX1A (delN, 89–217), pCMViR-3xFLAG-PRRX1A (delN, 167–217), pLEX307-3xTOP2A (Full-length)) was transfected into lentiX293T cells using PEI-MAX reagent (Polysciences, USA). After culturing for 48 h, a DDDDK-tagged or a HA-tagged Protein Magnetic Purification Kit (MBL, Japan) was used to purify recombinant proteins according to the manufacturer’s instructions. Purified proteins were mixed with 500 μL of lysis buffer (150 mM NaCl, 10 mM Tris-HCl (pH 7.4), 30 mM sodium pyrophosphate, 10 mM NaF, 1% NP-40, 0.5 mM EDTA and one Complete Protease Inhibitor Mixture Tablet TM (Roche, Brussels, Belgium)) containing 20 µL of anti-FLAG magnetic beads (MBL, Japan) and rotated at 4 °C for 24 h. The beads were washed three times with 10 mM PBS and 0.05% Tween-20 and incubated with 45 µL of 1 mg/mL DDDDK peptide solution (MBL, Japan) to elute FLAG-tagged proteins.

### Additional assays

Details of cell proliferation assay, wound healing assay, transwell assay, western blotting and quantitative real-time reverse transcription-PCR have been documented in the supplementary section (Supplementary Materials & methods).

### Structural prediction

AlphaFold2 (v. 2.2; AF2) was used to predict the structure of TOP2A. Default values were used for all parameters. FoldDock [26], an extension of AF2, was used to predict the structure of the PRRX1-TOP2A complex. For each structure prediction, the database was specified as the shared AF2 database (v2.1) on the supercomputer ‘Flow’ at the Information Technology Center, Nagoya University. PyMOL (2.5.0 Open-Source) was used to draw the predicted structures.

### Statistical analysis

Data analysis was performed using Prism 8 (GraphPad Software, San Diego, CA, USA). Kaplan‒Meier plots and log-rank tests were used to evaluate survival statistics. All data were acquired by performing three independent biological replicates for each experiment and are presented as the means ± SEMs. Statistical significance was determined using a two-tailed *t* test or unpaired one-way or two-way ANOVA with Tukey’s post hoc analysis, as appropriate. No statistical sample size calculations were performed. However, with a sample size of 23 MPNST patients and a two-sided significance level of *P* < 0.05, the post hoc power was calculated to be 0.68. Power calculations for in vivo experiments were based on our previous study [21].

## Results

### PRRX1 is expressed in various types of human sarcoma tissues

**Fig. 1:**
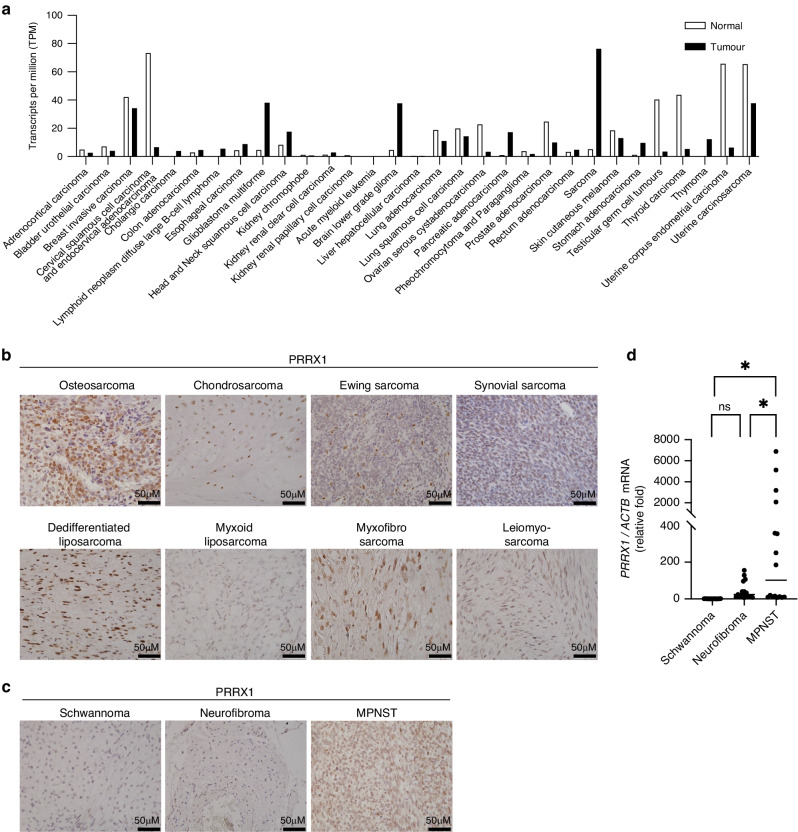
Expression of PRRX1 in human sarcoma tissues. **a** Expression profile of PRRX1 mRNA in tumour samples. Data were obtained from the GEPIA Platform (http://gepia.cancer-pku.cn). **b**, **c** Immunostaining of PRRX1 in human sarcoma tissues. Sections derived from human sarcoma tissues were stained with PRRX1 and representative images are shown. **d** qPCR analysis of PRRX1 in human schwannoma, neurofibroma and malignant peripheral nerve sheath tumour (MPNST) tissues. Total RNA was extracted to compare the expression level of PRRX1 mRNA. All values were normalised to ACTB mRNA levels (*n* = 3). Data are presented as the means ± SEMs. * *p* < 0.05.

To compare the expression level of *PRRX1* between normal and tumour tissues, we used the GEPIA Platform (http://gepia.cancer-pku.cn), which can analyse The Cancer Genome Atlas (TCGA) and Gene Tissue Expression (GTEX) databases. Notably, sarcoma tissues expressed a significantly higher level of *PRRX1* than normal or other tumour tissues (Fig. 1a). We collected human sarcoma tissue samples for immunohistochemical analysis and found that these sarcomas expressed *PRRX1* (Fig. 1b). Interestingly, the expression of *PRRX1* in MPNST was higher than that in schwannoma or neurofibroma at both the protein and RNA levels (Fig. 1c, d). MPNST can arise as a result of the malignant transformation of benign tumours, such as schwannoma or neurofibroma. These results indicate that *PRRX1* is expressed in various human sarcomas and that its level may increase during malignant progression.

### PRRX1 levels are positively correlated with poor prognosis in MPNST patients

**Fig. 2:**
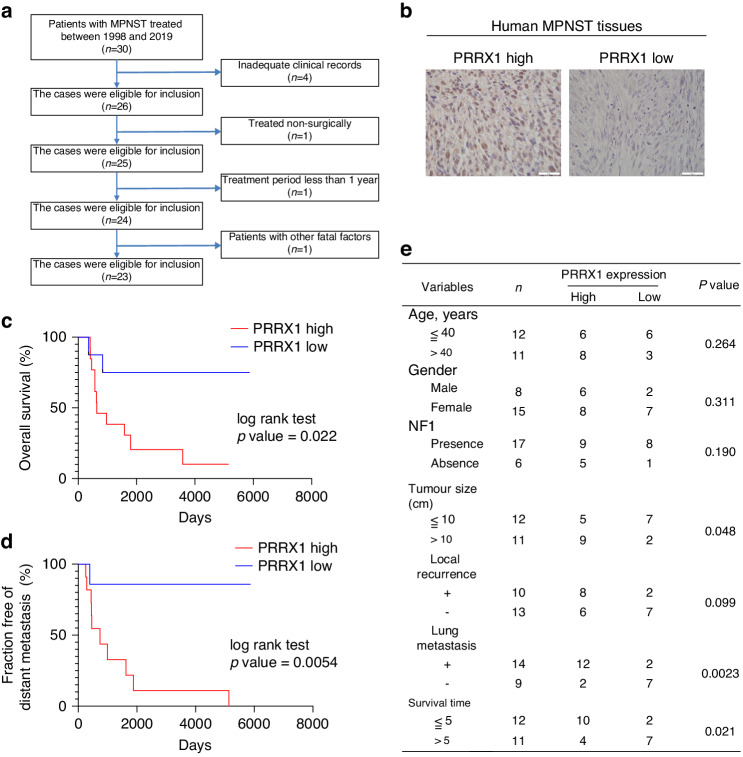
Expression of PRRX1 in human MPNST tissues. **a** Criteria for choosing human MPNST tissue samples. **b** Immunostaining of PRRX1 in human MPNST tissues. Sections derived from human MPNST were stained with PRRX1 and representative images of PRRX1-low or PRRX1-high tumours are shown. **c**, **d** Comparison of overall survival or distant metastasis between PRRX1-low and PRRX1-high MPNST patients. The Kaplan–Meyer survival curve demonstrates significant differences (log-rank test, *p* < 0.05). **e** Correlation of PRRX1 levels with patient clinical and pathological characteristics.

Based on the criteria listed in Fig. 2a, we analysed 23 MPNST patients and subdivided them into high- (*n* = 14, 61%) and low-expression *PRRX1* groups (*n* = 9, 39%) (Fig. 2b). Interestingly, the *PRRX1* expression level was significantly associated with 5-year overall survival (high *vs*. low: 10% *vs*. 75%, *P* < 0.05) and the incidence of distant metastasis (high *vs*. low: 100% *vs*. 14%, *P* < 0.05) (Fig. 2c, d). Comparison of *PRRX1* expression levels between patients with or without lung metastasis demonstrated that *PRRX1*-high patients more frequently belonged to the lung metastasis group (85.7% *vs*. 22.2%, *P* < 0.01). Furthermore, the *PRRX1* expression level was significantly higher in patients who survived less than 5 years than in those who survived more than 5 years. No significant correlation was observed between *PRRX1* expression and age, sex, or local disease recurrence (Fig. 2e). These results demonstrate that the expression of *PRRX1* is associated with the malignancy of MPNST.

### PRRX1 knockdown decreased the proliferation and tumorigenicity of HS-PSS

**Fig. 3:**
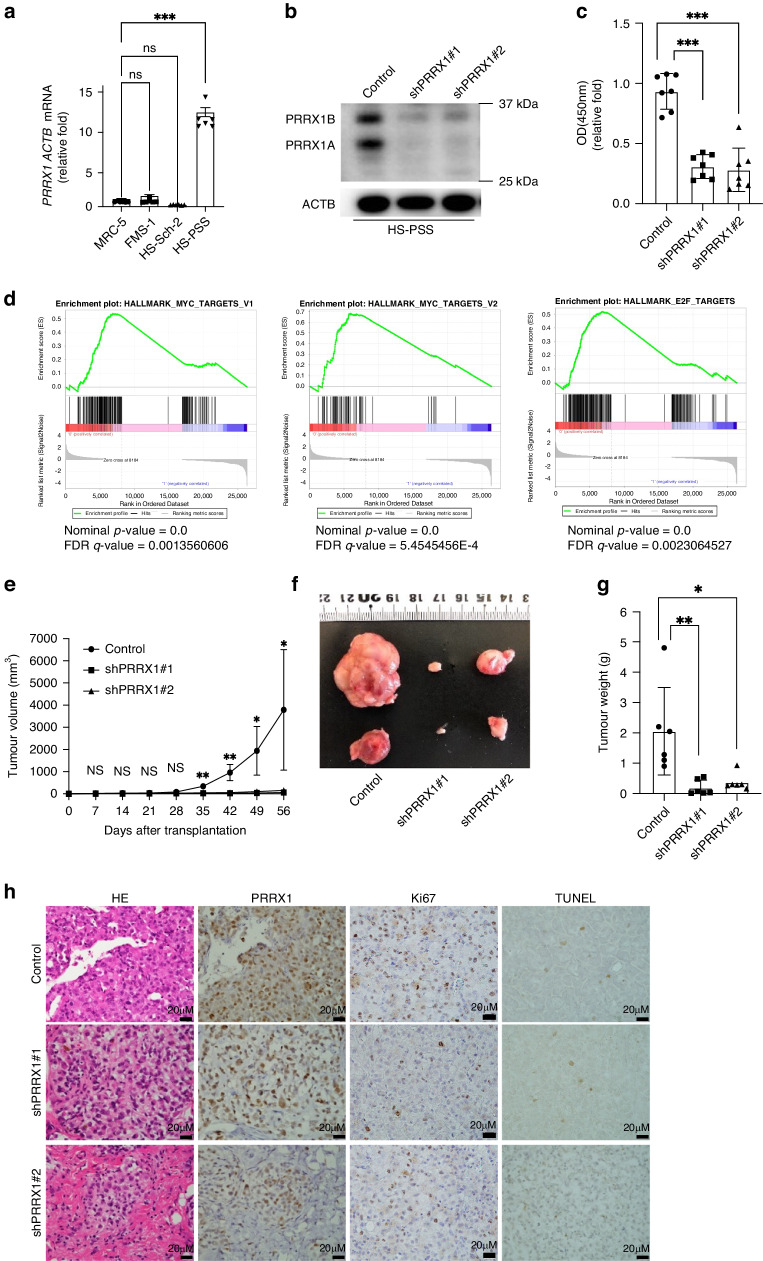
Effects of PRRX1 knockdown on the proliferation or tumorigenesis of human MPNST cells. **a** Comparison of PRRX1 mRNA among human MPNST cell lines by qPCR analysis. Total RNA was extracted from human foetal lung fibroblasts (MRC-5) or the human MPNST cell lines FMS-1, HS-Sch-2 and HS-PSS to compare the expression level of PRRX1 mRNA. All values were normalised to ACTB mRNA levels (*n* = 3). **b** Western blot analysis after PRRX1 knockdown. HS-PSS cells were infected with lentivirus encoding each shPRRX1 clone and total cell lysates were extracted to compare the expression level of PRRX1. **c** Comparison of proliferative capacity after PRRX1 knockdown by WST-8 assay. (*n* = 7, three independent experiments). **d** Comparison of the RNA transcriptome after PRRX1 knockdown. RNA-seq analysis of HS-PSS/Control and HS-PSS/shPRRX1#1 and #2 was performed and data were compared by gene set enrichment analysis (GSEA). Genes upregulated in HS-PSS/Control are clustered on the left (*n* = 2, two independent experiments). **e**, **f**, **g** Comparison of tumour volume and weight after PRRX1 knockdown. Tumour volume was measured at each indicated time point after subcutaneous transplantation into NOD-SCID mice. At 56 days after transplantation, mice were sacrificed and the weights of the developed tumours were measured (*n* = 6, three independent experiments). **h** Immunohistological analysis of developed xenograft tumours. Tumour sections were stained with hematoxylin and eosin (H&E), immunostained for PRRX1 or Ki-67, or stained by TUNEL. Representative photomicrographs are shown. Data are presented as the means ± SEMs. * *p* < 0.05; ** *p* < 0.01; *** *p* < 0.001.

To investigate the function of PRRX1 in human MPNST, we examined its expression in several human MPNST cell lines using qPCR (HS-PSS, FMS-1 and HS-Sch-II). *PRRX1* expression was higher in HS-PSS than in the other cell lines; thus, HS-PSS was used in further experiments (Fig. 3a, S1A). To suppress the function of *PRRX1*, two shPRRX1 lentiviruses were designed to knockdown *PRRX1*. After HS-PSS cells were infected with empty (control) or shPRRX1 lentiviruses, the downregulation of *PRRX1* was confirmed by western blot analysis (Fig. 3b). Analysis of cell proliferation and migration after *PRRX1* knockdown demonstrated that the proliferative and migratory activity of shPRRX1 cells was lower than that of control cells (Fig. 3c, S1B–D). The inhibitory effect of *PRRX1* knockdown on proliferation was also confirmed using another human MPNST line, FMS-1 (Fig. S2A–C). To analyse the systematic effects of *PRRX1* knockdown on the RNA transcriptome, RNA-seq or GSEA was performed. As shown in Fig. 3d, proliferation-associated gene sets, including MYC target V1, MYC target V2 and E2F, were downregulated in *PRRX1*-knockdown cells. The results of the GSEA analysis using a different database to analyse the systematic effects of *PRRX1* knockdown resulted in reduced cell motility (Fig. S3A–C). In addition, the effect of PRRX1 knockdown was evaluated with well-known EMT-related markers (CDH2, TWIST), with both results being suppressed. However, it was not clear in the characteristics of the MPNST cell line, so further experiments are needed to determine how PRRX1 functions in EMT in the future (Fig. S4A, B). To determine the effect of *PRRX1* knockdown on the tumorigenicity of HS-PSS cells, xenograft transplantation assays were next performed. The growth of shPRRX1 tumours was significantly suppressed (Fig. 3e–g). Additionally, tumours derived from shPRRX1 cells showed lower expression of *PRRX1* and Ki-67 than those derived from control cells (Fig. 3h, S4C, D). The cell death ratio, as assessed by a TUNEL assay, was not significantly different between control cells and shPRRX1 cells (Fig. 3h, S4E). Since none of the mice showed lung metastasis, it was not possible to assess the correlation between metastasis and *PRRX1* expression. These results demonstrate that PRRX1 promotes the proliferation and tumorigenicity of human MPNST cells.

### PRRX1 interacts directly with TOP2A in human MPNST cells

To identify the proteins that interact with PRRX1, we introduced the doxycycline-inducible 3xFLAG-PRRX1A expression cassette into HS-PSS cells to establish an HS-PSS/3xFLAG-PRRX1A line. FLAG-tagged PRRX1A was detected in HS-PSS/3xFLAG-PRRX1A lysate by treating cells with doxycycline (Fig. S5A). HS-PSS cells overexpressing *PRRX1A* showed increased migration ability and changed to a mesenchymal-like cell shape (Fig. S5B, C). Same experiments were performed with *PRRX1B* and similar properties to *PRRX1A* were confirmed (Fig. S5D–F). Both benign and malignant tumours resulted in higher expression of *PRRX1B*, but in malignant tumours, the relative change in expression of *PRRX1A* was higher (Fig. S5G).

**Fig. 4:**
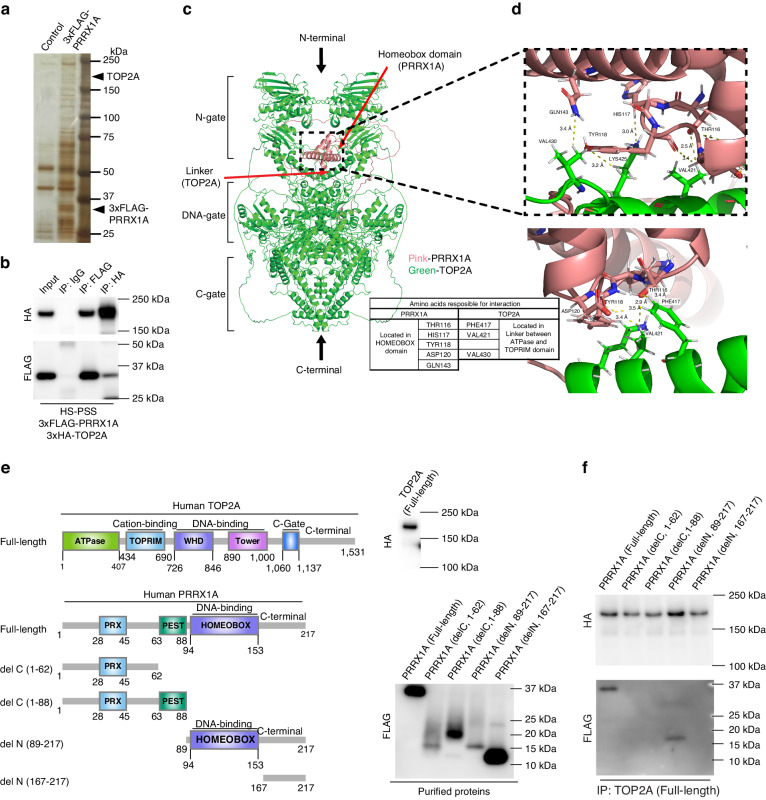
Identification of the PRRX1–TOP2A interaction in human MPNST cells. **a** Silver staining after immunoprecipitation of 3xFLAG-PRRX1A. HS-PSS/3xFLAG-PRRX1A cells were treated with 1 µg/mL doxycycline (DOX) and then cell lysates were extracted to immunoprecipitate 3xFLAG-PRRX1A and its associated proteins. The cell lysate of HS-PSS was used to detect nonspecific signals. **b** Confirmation of the PRRX1-TOP2A interaction by immunoprecipitation. After treating HS-PSS/3xFLAG-PRRX1A/3xHA-TOP2A cells with 1 µg/mL DOX, cell lysates were extracted to immunoprecipitate 3xFLAG-PRRX1A or 3xHA-TOP2A. Input and each immunoprecipitated sample were detected with an anti-FLAG or an anti-HA antibody. **c** Structure binding prediction of PRRX1A and TOP2A dimer by FoldDock. PRRX1A is shown in pink and TOP2A in green. **d** Enlarged view of predicted binding sites. Interatomic distances were measured by PyMOL and amino acid residues at possible hydrogen bonding distances were plotted in the stick bond model. Predicted hydrogen bonds are indicated by yellow dotted lines. **e** Schematic drawing of each 3xFLAG-PRRX1A deletion construct or 3xHA-TOP2A (full-length). Each expression vector was transfected into HEK293T cells and each protein was purified from cell lysates. **f** Cell-free immunoprecipitation using purified 3xFLAG-PRRX1A deletion constructs and 3xHA-TOP2A (full-length). Purified PRRX1A deletion constructs and 3xHA-TOP2A (full-length) were conjugated in lysis buffer and immunoprecipitation with anti-FLAG antibody was performed.

Whole-cell lysates of HS-PSS/3xFLAG-PRRX1A were immunoprecipitated with anti-FLAG antibody, visualised with silver staining (Fig. 4a) and analysed by shotgun mass spectrometry, which revealed that 93 proteins were confirmed. Among these, topoisomerase 2 alpha (TOP2A) was identified as a PRRX1 interaction protein. We next established an HS-PSS/3xFLAG-PRRX1A/3xHA-TOP2A line that expressed FLAG-tagged PRRX1A and HA-tagged TOP2A doxycycline treatment and confirmed the PRRX1-TOP2A interaction by co-immunoprecipitation with anti-FLAG or anti-HA antibodies (Fig. 4b). To verify the PRRX1-TOP2A interaction from the protein structure, AlphaFold2 (v. 2.2; AF2) analysis was used to predict the structure of TOP2A. Based on the previously reported structure (PDB_ID: 6ZY8) [27] determined by cryo-electron microscopy (Cryo-EM), the structure prediction of TOP2A and the PRRX1-TOP2A complex could be validated (Fig. S6A, B). Next, PyMOL was used to model the TOP2A dimer and verify the stoichiometry of the interaction between PRRX1 and the TOP2A dimer (Fig. 4c). The PyMOL results suggested that the PRRX1 monomer is bound to the TOP2A dimer. The binding sites were predicted to be between the HOMEOBOX domain of PRRX1 and the linker sites of the ATPase and TOPRIM of TOP2A, which are bound by seven hydrogen bonds (Fig. 4d). To confirm these predictions, we designed a series of PRRX1A deletio77n constructs, as described in Fig. 4e and generated expression vectors encoding 3xFLAG-PRRX1A Full-length/deletion constructs or 3xHA-TOP2A Full-length. Each plasmid was introduced into HEK293T cells and proteins were purified to test the protein–protein interaction under cell-free conditions. As shown in Fig. 4f, western blot analysis detected each purified protein and full-length PRRX1A or del N (89–217) coimmunoprecipitated with full-length TOP2A. The fact that delC (1–88) and del N (167–217) did not coimmunoprecipitate suggests that the PRRX1-TOP2A interaction occurs at del N (89–217) of PRRX1. Remarkably, the results were in close agreement with the structural predictions of AF2. These results demonstrate that the DNA-binding domain (HOMEOBOX) of PRRX1 directly interacts with TOP2A.

### Etoposide inhibited the PRRX1–TOP2A interaction

**Fig. 5:**
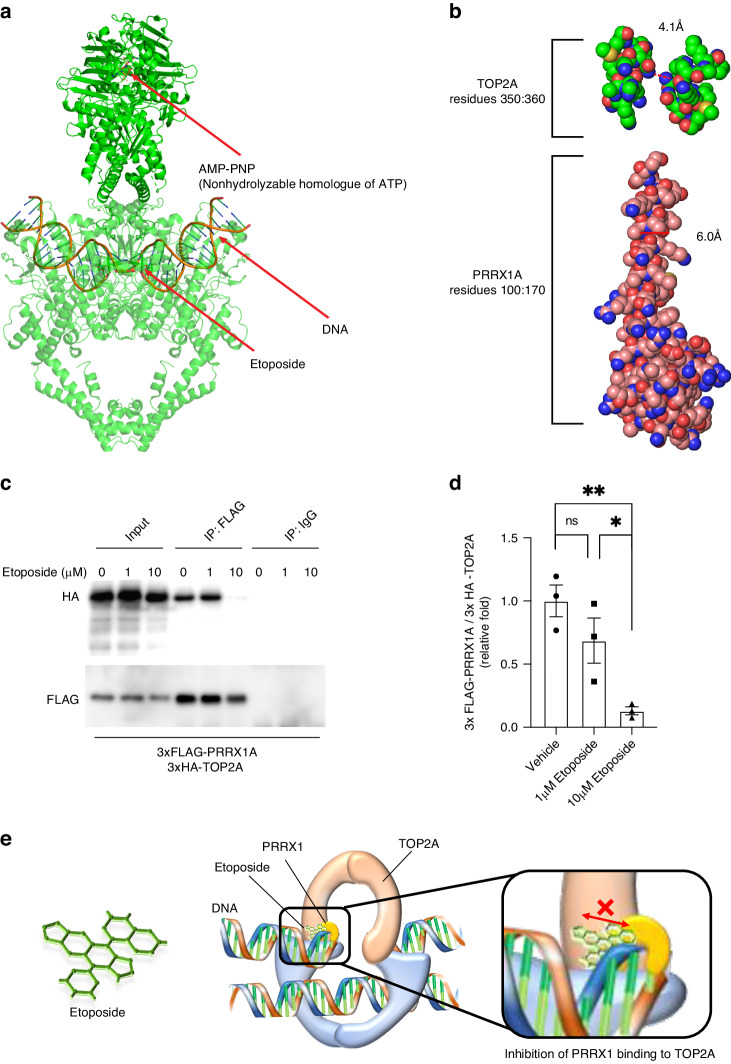
Effect of the TOP2A inhibitor etoposide on the PRRX1A–TOP2A interaction. **a** Structure of TOP2A in the presence of etoposide and ATP (6ZY8). To allow the binding site of TOP2A to be seen, the DNA-gate and C-gate of TOP2A are translucent. **b** At 6ZY8, the width of the narrowest PRRX1A binding site was measured by PyMOL. Amino acid residues in the vicinity of the measurement site were plotted using a space-filling model (PRRX1A: residues 100:170, TOP2A: residues 350:360). The line segments used for the measurements are indicated by red dotted lines. **c** Inhibition of the PRRX1A–TOP2A interaction by etoposide. After treating HS-PSS/3xFLAG-PRRX1A/3xHA-TOP2A cells with 1 µg/mL DOX and DMSO (vehicle) or each indicated concentration of etoposide, cell lysates were extracted to immunoprecipitate 3xFLAG-PRRX1A. Input and each immunoprecipitated sample were detected with anti-FLAG or anti-HA antibody. **d** Semiquantitative analysis of HA-TOP2A levels. The western blot band intensities were measured using ImageJ software. HA-TOP2A protein levels were normalised to FLAG-PRRX1A levels. The intensity (mean ± standard error) was normalised to control values. Statistical significance was determined using unpaired one-way ANOVA with Tukey’s post hoc analysis. **e** Schematic illustration of the proposed mechanism by which the PRRX1-TOP2A interaction is inhibited by etoposide. The action of etoposide on TOP2A inhibits binding to PRRX1. Inhibition of the interaction between PRRX1 and TOP2A by etoposide stops the progression of DNA cleavage, causing double-strand DNA breaks and etoposide exerts its anticancer effect. Data are presented as the means ± SEMs. * *p* < 0.05; ** *p* < 0.01.

To further investigate the PRRX1–TOP2A interaction, we examined the effect of etoposide, a TOP2A inhibitor, both from structural prediction and in vivo *experiments*. The TOP2A structure during DNA cleavage was predicted using AF2 when AMP-PNP, a nonhydrolyzable homologue of ATP and etoposide were acting on (Fig. 5a). In this case, the binding site of PRRX1 in TOP2A is 4.1 Å and even the narrowest PRRX1 α-helix is 6.0 Å, resulting in inhibition of PRRX1–TOP2A binding (Fig. 5b). To confirm this prediction, we treated HS-PSS/3xFLAG-PRRX1A/3xHA-TOP2A cells with 1 or 10 µM etoposide and found that 10 µM etoposide reduced the amount of TOP2A that coimmunoprecipitated with 3xFLAG-PRRX1A (Fig. 5c, d). These results suggest that etoposide may inhibit the direct binding site of PRRX1 and TOP2A. (Fig. 5e)

### The PRRX1–TOP2A interaction is a malignant factor in human MPNST

To determine the effect of the PRRX1–TOP2A interaction on tumour malignancy, we analysed clinical samples. When the expression profile of *TOP2A* mRNA in human tumour or normal tissues was analysed using the GEPIA Platform, various tumour tissues, including sarcoma, showed higher levels of *TOP2A* expression than normal tissues (Fig. S7A, B). The *TOP2A* expression level was significantly associated with 5-year overall survival (Fig. S8A, high *vs*. low: 0% *vs*. 100%, *P* < 0.05) and distant metastasis (Fig. S8B, high *vs*. low: 0% *vs*. 100%, *P* < 0.05). Immunohistochemical analysis revealed that human MPNST tissues were stained with PRRX1 and TOP2A, but healthy bone tissues were not (Fig. S8C). Interestingly, there was a positive correlation between PRRX1 and TOP2A levels in human MPNST tissues (Fig. S8D, E). To test the cooperative effect of TOP2A on PRRX1-promoted migration, we designed two shTOP2A lentiviruses. HS-PSS/3xFLAG-PRRX1A cells were infected with empty or shTOP2A lentiviruses and reduced *TOP2A* expression in knockdown cells was confirmed by western blot analysis (Fig. S8F). Scratch assays showed that the effect of *PRRX1* overexpression was counteracted by suppressing *TOP2A*. (Fig. S8G, H).

**Fig. 6:**
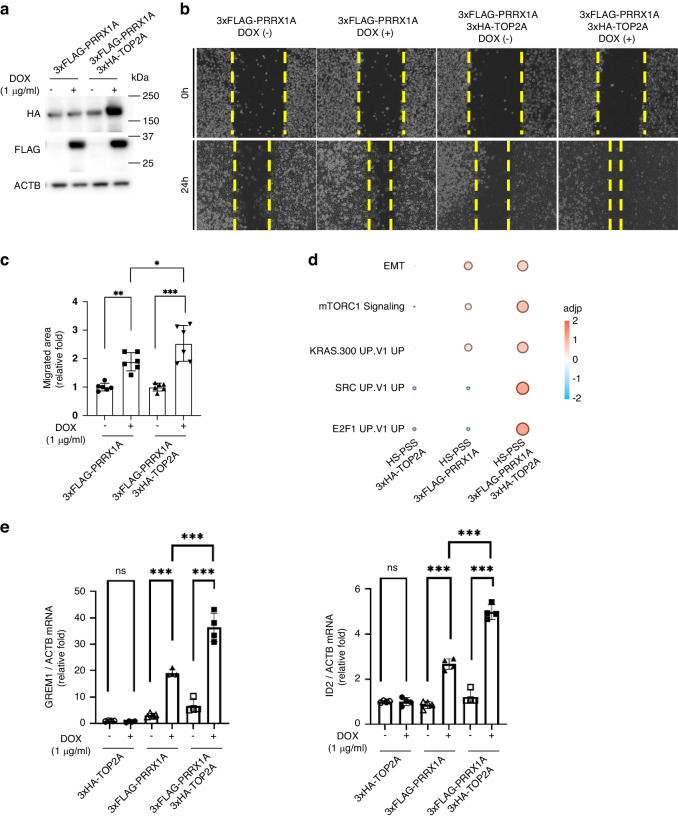
The PRRX1–TOP2A interaction plays critical roles in migration and oncogenic signalling. **a** Western blot analysis to detect 3xFLAG-PRRX1A and 3xHA-TOP2A. HS-PSS/3xFLAG-PRRX1A or HS-PSS/3xFLAG-PRRX1A/3xHA-TOP2A cells were treated with 1 µg/mL DOX for 1 day and then total cell lysates were extracted. **b**, **c** Comparison of migration capacity by wound healing assay. HS-PSS/3xFLAG-PRRX1A or HS-PSS/3xFLAG-PRRX1A/3xHA-TOP2A cells were treated with 1 µg/mL DOX for 1 day and then the assay was started (*n* = 6, three independent experiments). **d** Comparison of GSEA results performed on MSigDB for HS-PSS/3xHA-TOP2A, HS-PSS/3xFLAG-PRRX1A or HS-PSS/3xFLAG-PRRX1A/3xHA-TOP2A cells. In the significantly enriched oncogenic-related pathways, the size of the circle indicates the number of differentially expressed genes and the colour of the circle indicates the adjusted *p* value normalised by the z score. **e** Comparison of EMT-related genes (GREM1, ID2) mRNA among HS-PSS/3xHA-TOP2A, HS-PSS/3xFLAG-PRRX1A or HS-PSS/3xFLAG-PRRX1A/3xHA-TOP2A cells by qPCR analysis. Each cell was treated with 1 µg/mL DOX for 1 day and then the assay was started (*n* = 4, two independent experiments). All values were normalised to ACTB mRNA levels. Data are presented as the means ± SEMs. * *p* < 0.05; ** *p* < 0.01; *** *p* < 0.001.

Next, after confirming the expression of 3xFLAG-PRRX1A and 3xHA-TOP2A by western blot analysis (Fig. 6a), we compared the migration of HS-PSS/3xFLAG-PRRX and HS-PSS/3xFLAG-PRRX1A/3xHA-TOP2A cells, which revealed that coexpression of 3xHA-TOP2A in 3xFLAG-PRRX1A cells cooperatively promoted cell migration (Fig. 6b, c). Total RNA was extracted from HS-PSS/3xHA-TOP2A, HS-PSS/3xFLAG-PRRX1A, or HS-PSS/3xFLAG-PRRX1A/3xHA-TOP2A cells and RNA-sequencing analysis was performed to compare their RNA transcriptomes. Analysis by enrichR showed enrichment of genes involved in cancer-related pathways, as shown in the Supplemental Table S5, in cells overexpressing 3xFLAG-PRRX1A. In particular, genes involved in tumour progression, such as epithelial-mesenchymal transition (EMT) and mTORC1 signalling, were found to be significantly enriched (Fig. 6d). EMT-related genes (*GREM1, ID2*) in the pathway enriched by RNA-seq in Fig. 6d were confirmed by qPCR. The results showed that PRRX1A cooperatively with TOP2A increased the expression of EMT-related genes (*GREM1, ID2*) (Fig. 6e). [28–31]

These results indicate that the PRRX1–TOP2A interaction may be involved in regulating tumour malignancy by promoting metastasis or oncogenic signalling. In the mRNA expression profiles analysed using the GEPIA Platform, *PRRX1* and *TOP2A* were highly expressed in glioblastoma and pancreatic cancer as well as MPNST (Fig. 1a and Fig. S7A). Therefore, we analysed protein levels in MPNST, glioblastoma, pancreatic cancer and small cell lung cancer cell lines and confirmed the expression of *PRRX1* and *TOP2A* (Fig. S7B). These results indicate that *PRRX1* and *TOP2A* are expressed in a variety of human malignant tumours and that their interaction may occur in any malignancy.

## Discussion

In this study, we showed that *PRRX1* was highly expressed in various types of human sarcoma tissues and might contribute to malignancy in MPNST patients by enhancing cell motility. Corroborating our results, several groups have reported that high expression of *PRRX1* not only promotes tumour cell malignancy, but also plays an important role in cancer associated fibroblasts (CAFs) in promoting tumorigenesis, metastasis and cancer recurrence. [7, 8, 21, 32, 33]. Indeed, *PRRX1* was highly expressed in CAFs of various sarcomas and less so in benign tumours such as schwannoma and neurofibroma (Fig. S9). During development, *Prrx1* is expressed in limb bud mesenchyme and neural crest cells [34–37]. MPNST is postulated to originate from Schwann cell lineages, which are generated from neural crest cells [38, 39]. EMT for acquiring high migration ability is characteristic feature of neural crest cells and this property can also be used by cancer cells to promote malignancy or establish metastasis [40, 41]. These results indicated that PRRX1 may promote dedifferentiation toward a neural crest-like state and induce the malignancy of human MPNST.

Here, we identified a direct interaction between PRRX1 and TOP2A, revealing a mechanism by which high *PRRX1* and *TOP2A* expression is associated with poor prognosis in MPNST patients. TOP2A is traditionally known as a duplex DNA ‘strand-passage’ enzyme that resolves entanglements and relieves torsional stress of DNA by the production of DNA double-stranded breaks and its upregulation is associated with the malignancy of several tumours [42–44]. In this study, TOP2A cooperatively increased the expression of EMT gene sets whose expression was upregulated by *PRRX1*, suggesting that TOP2A may affect PRRX1 binding to specific promoter regions of genes involved in oncogenesis. In addition, tissues of several other cancers, including invasive breast carcinomas with the BRCA mutation, glioblastoma multiforme and pancreatic adenocarcinoma, also expressed high levels of *PRRX1* and *TOP2A* (Fig. 1a and Fig. S7A), indicating that functional inhibition of the PRRX1-TOP2A interaction might provide an alternative strategy to direct inhibition of TOP2A, which has severe side effects in vivo [45], for the therapy of a broad spectrum of human cancers.

TOP2A inhibitors such as doxorubicin and etoposide have demonstrated some efficacy against MPNST in phase II clinical trials [19, 46, 47]. In particular, etoposide has been shown to be a potential first-line treatment, but its therapeutic application is limited due to severe side effects on normal tissues [48, 49]. In this study, we showed that a high concentration of etoposide may exhibit one of its anticancer effects by inhibiting the PRRX1–TOP2A interaction in vitro (Fig. 5c, d), indicating that the interaction is a potential therapeutic target. Human MPNST cell lines express higher levels of *TOP2A* than human neurofibroma cell lines and show higher sensitivity to doxorubicin [50]. These results suggest that a combination therapy comprising a PRRX1–TOP2A interaction inhibitor and a TOP2A inhibitor might be more effective than using a TOP2A inhibitor alone or may reduce the amount of TOP2A inhibitor needed, thus reducing the severity of TOP2A inhibitor side effects. In addition, our prediction of the mechanisms underlying the PRRX1-TOP2A interaction (Fig. S10) may provide further understanding of this novel therapeutic target. A limitation of the present study was that the association between increased *PRRX1* expression and tumour malignancy progression has not been sufficiently proven to be applicable to other malignancies, so further experiments are needed.

## Conclusions

Our results demonstrate that PRRX1 is a potential therapeutic target for MPNST. Identification of the PRRX1-TOP2A interaction as one of the mechanisms involved in tumour malignancy has clarified the pathophysiological background of MPNST and raised the prospect of developing novel therapeutic strategies for malignant tumours with poor prognosis, including MPNST.

### Supplementary information

Supplementary Materials and methods

Supplementary figure

Table S1

Table S2

Table S3

Table S4

Table S5

## Data Availability

*PRRX1* or *TOP2A* mRNA expression profiling data in tumour samples were obtained from the GEPIA Platform (http://gepia.cancer-pku.cn). The raw and processed RNA-seq data were deposited in the NCBI GEO database under accession number GSE 214899. All other data associated with this study are presented in the paper or the Supplementary Materials. The additional datasets used and/or analysed during the current study are available from the corresponding author upon reasonable request.
