# Supplementary material for: PRRX1-TOP2A interaction is a malignancy-promoting factor in human malignant peripheral nerve sheath tumours
Source: Br J Cancer. 2024 Mar 6;130(9):1493–504. doi: 10.1038/s41416-024-02632-8 (PMC11058259; doi:10.1038/s41416-024-02632-8)
Supplement: Supplementary file 1 — Supplementary Materials and methods [file 41416_2024_2632_MOESM1_ESM.docx]

**Supplementary Materials & methods**

*Cell culture*

FMS-1 cells were maintained in RPMI-1640 medium (Sigma, USA) supplemented with 15% heat-inactivated fetal bovine serum (FBS; Hyclone, Australia), penicillin (100 U/mL), and streptomycin (100 mg/mL) (Thermo Fisher Scientific). All other cells were maintained in Dulbecco’s modified Eagle’s medium (DMEM; FUJIFILM Wako., Japan) supplemented with 10% FBS, penicillin (100 U/mL), and streptomycin (100 mg/mL). All cells were cultured at 37°C in a humidified atmosphere containing 5% CO_2_.

*Cell proliferation assay*

Cells were plated onto 96-well plates at 2 × 10^3^ cells per well in a final volume of 100 μL per well. Cell viability was determined using the cell proliferation reagent WST-8 (Dojindo, Tokyo, Japan) according to the manufacturer’s protocol. For comparison of proliferative capacity, cells were cultured for 7 days. WST-8 substrate (10 μL) was added to each well and incubated for 2 h at 37˚C in 5% CO_2_. Absorbance at 450 nm was measured using a microplate reader (Bio-Rad Laboratories) and the number of cells was counted manually at the same time. Each experiment was performed in triplicate.

*Wound healing assay*

A scratch wound healing assay was used to examine cell mobility characteristics. Briefly, cells (2 × 10^5^ cells/well) were grown in a 24-well plate until confluent. Cell monolayers were scratched (wounded) using a sterile 200 μL pipette tip, and PBS was used for washing and removing cell debris. After 24 h, migrating cells were monitored and photographed under phase-contrast microscopy. Image J software was used to quantify the relative wound size. Cell mobility inhibition (%) was calculated as the new scratch width / original scratch width × 100%. Experiments were repeated three times.

*Transwell assay*

Cell invasion and migration were examined with 24-well BD BioCoat invasion chambers. Matrigel matrix (BD, USA) was used for invasion assays. A total of 1 × 10^5^ cells were suspended in 500 μL DMEM without FBS and added to the upper chamber. DMEM with 10% FBS was added to the lower chamber. After incubation for 24 h, the cells on the upper surface of the filter were completely removed by wiping with cotton swabs. The filters were fixed in methanol and stained with Hemacolor^®^ solution 3 (Merck, Germany). The filters were then mounted onto slides, and the cells on the lower surfaces were counted in six randomized high-power fields.

*Immunofluorescence (IF) analyses*

To perform double immunofluorescence staining, clinical samples of MPNST and normal bone tissue were prepared and mounted on slides. The slides were processed and blocked in the manner described for IHC. Double immunofluorescence staining was performed by incubating deparaffinized sections overnight at 4°C with a mixture of anti-PRRX1 monoclonal (1:400 dilution; OTI6A4; Thermo fisher Scientific) and anti-TOP2A rabbit polyclonal (1:400 dilution; 20233-1-AP, Proteintech, USA) antibodies. After washing with PBS, sections were incubated for 1 h in the dark at room temperature in a mixture of the Alexa-647 and Alexa-488 (1:400 dilution; Thermo fisher Scientific). Slides were then washed in PBS and counterstained by incubation for 5 min with DAPI (5 mg/ml of an aqueous solution diluted 1:2000 in blocking buffer). The slides were then mounted and analyzed under a confocal fluorescence microscope (Keyence BZ-X710, Kyoto, Japan).

*Western blotting*

Western blotting was performed as previously described[1]. The following antibodies were used; rabbit polyclonal anti-PRRX1 (HPA-051084, 1:2000 dilution; Sigma Aldrich), mouse monoclonal anti-PRRX1 (MA5-26579, 1:2000 dilution; Thermo fisher Scientific, and ZRB2165, 1:2000 dilution; Sigma Aldrich), anti-TOP2A (UM870042, 1:2000 dilution; OriGene Technologies, Jiangsu, China), anti-FLAG (M185-3MS, 1:2000 dilution; Sigma Aldrich), anti-HA (M180-3, 1:2000 dilution; MBL, Nagoya, Japan), and mouse monoclonal anti-Beta Actin (sc4778, 1:2500 dilution; Santa Cruz Biotechnology, Santa Cruz, CA, USA).

*Quantitative real-time reverse Transcription-PCR*

Cells were lysed in Buffer RLT (QIAGEN), and RNA isolation was performed using the RNeasy Mini Kit (QIAGEN). Quantitative real-time PCR was performed using specific primers. The sequences of primers used are listed in Table S3. The relative mRNA expression was determined using the ΔCt method. The gene expression was normalized to ACTB. RT-PCR was performed using an AriaMX Real-Time PCR System (Agilent Technologies, Santa Clara, CA, USA). The cycle parameters were as follows: denaturation at 95°C for 30 s, annealing for 30 s at 62°C, and elongation for 30 s at 72°C. The expression level of each gene was calculated using the 2^−ΔΔCt^ method.

For absolute quantification, cDNA prepared from MSCs (Lonza Walkersville Inc, MD, USA) was subjected to PCR using each primer in table S4 as a standard sample, and five independent serial dilutions were made, and the standard curves were generated by plotting the Ct value against the logarithm of the quantity (copy number). In this study, PCR was performed using KOD FX Neo™ (Toyobo Corporation, Osaka, Japan). Each sample was tested on the same reaction plate as the standard samples and the respective copy number was measured using the equation derived from the standard curves to calculate the exact number of cDNA molecules. All RT-PCR reactions were performed in duplicate.

**Reference**

1. Joko, R., et al., *PRRX1 promotes malignant properties in human osteosarcoma.* Transl Oncol, 2021. **14**(1): p. 100960.
