# Supplementary material for: PRRX1-TOP2A interaction is a malignancy-promoting factor in human malignant peripheral nerve sheath tumours
Source: Br J Cancer. 2024 Mar 6;130(9):1493–504. doi: 10.1038/s41416-024-02632-8 (PMC11058259; doi:10.1038/s41416-024-02632-8)
Supplement: Supplementary file 2 — Supplementary figure [file 41416_2024_2632_MOESM2_ESM.docx]

**Supplementary figures for**

**PRRX1-TOP2A interaction is a malignancy-promoting factor in human malignant peripheral nerve sheath tumors**

Shota Takihira^1,2^, MD, Daisuke Yamada^1^, PhD, Tatsunori Osone^1^, PhD, Tomoka Takao^1^, PhD, Masakiyo Sakaguchi^3^, PhD, Michiyuki Hakozaki^4^, PhD, Takuto Itano^2^, MD, Eiji Nakata^2^, PhD, Tomohiro Fujiwara^2^, PhD, Toshiyuki Kunisada^2^, PhD, Toshifumi Ozaki^2^, PhD, Takeshi Takarada^1*^, PhD

*^1^Department of Regenerative Science, Okayama University Graduate School of Medicine, Dentistry and Pharmaceutical Sciences, Okayama, 700-8558, Japan.*

*^2^Department of Orthopedic Surgery, Okayama University Graduate School of Medicine, Dentistry and Pharmaceutical Sciences, Okayama, 700-8558, Japan.*

*^3^Department of Cell Biology, Okayama University Graduate School of Medicine, Dentistry and Pharmaceutical Sciences, Okayama, 700-8558, Japan.*

*^4^Department of Orthopedic Surgery, Fukushima Medical University School of Medicine, Fukushima, 960-1295, Japan.*

* Takeshi Takarada, PhD

2-5-1 Shikata-cho, Kita-ku, Okayama 700-8558, Japan.

TEL: +81-86-235-7407

E-mail: takarada@okayama-u.ac.jp.


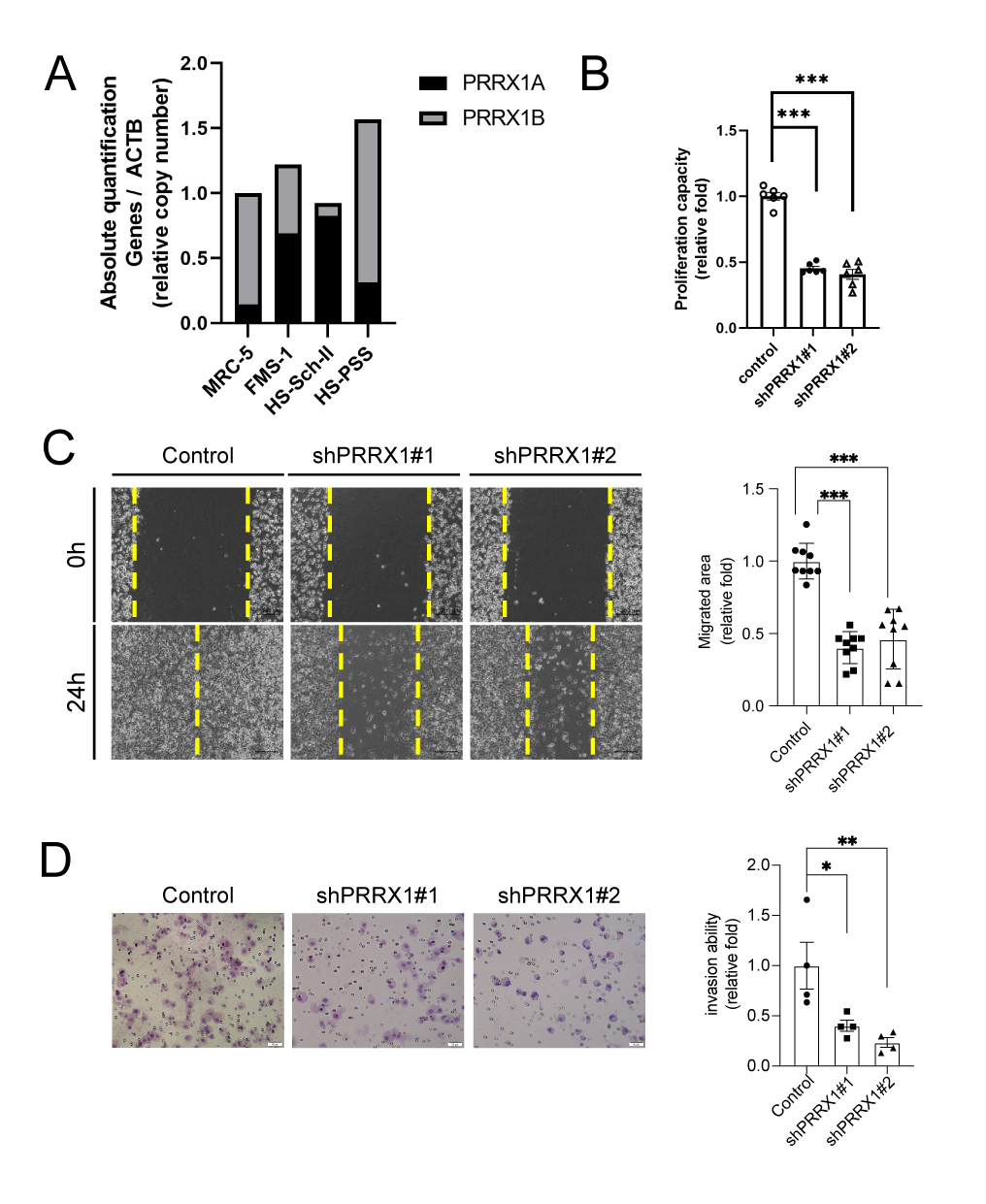


**Figure S1. PRRX1 knockdown suppressed the migration or invasion of HS-PSS cells**

(A) qPCR analysis for the absolute quantification (relative copy number to ACTB, reference gene) of PRRX1A or PRRX1B in MPNST cell lines. (B) Comparison of proliferative capacity after PRRX1 knockdown by cell counting assay (n = 6, three independent experiments). (C) Comparison of migration capacity after PRRX1 knockdown by wound healing assay. For the wound healing assay, the relative migration of HS-PSS/shPRRX1#1 or #2 cells was lower than that of HS-PSS/Control at 24 h after scratch wound (n = 9, three independent experiments). (D) Comparison of invasion capacity after PRRX1 knockdown by invasion assay. Transwell plates were used to assess the invasion. The number of migrated cells was significantly decreased in HS-PSS/shPRRX1#1 or #2 (n = 4, three independent experiments). Data are presented as the means ± SEMs. * p < 0.05; ** p < 0.01; *** p < 0.001.


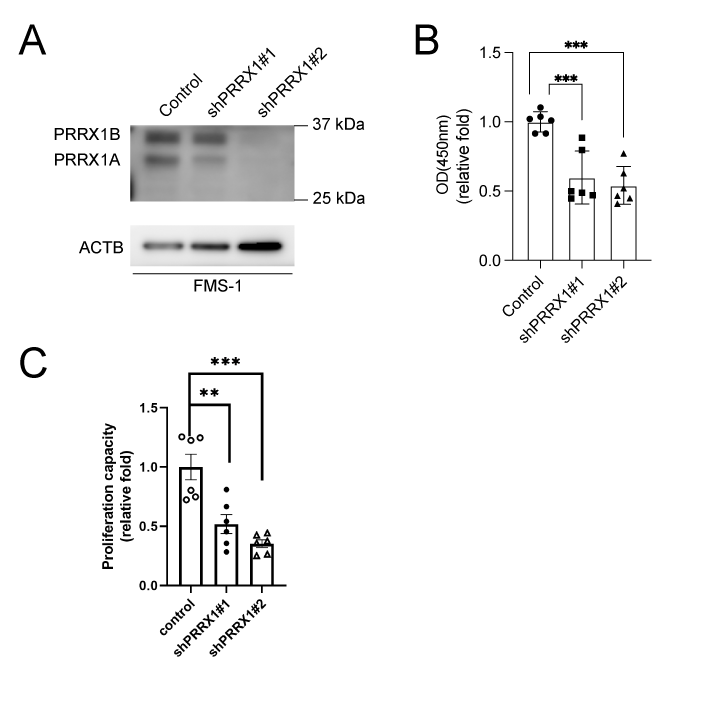


**Figure S2. PRRX1 knockdown suppresses proliferation of FMS-1**

(A) Western blot analysis after PRRX1 knockdown. FMS-1 cells were infected with lentivirus encoding each shPRRX1 clone, and total cell lysates were extracted to compare the expression level of PRRX1. (B) Comparison of proliferative capacity after PRRX1 knockdown by WST-8 assay. (n = 6, three independent experiments). (C) Comparison of proliferative capacity after PRRX1 knockdown by cell counting assay (n = 6, three independent experiments). Data are presented as the means ± SEMs. ** p < 0.01; *** p < 0.001.


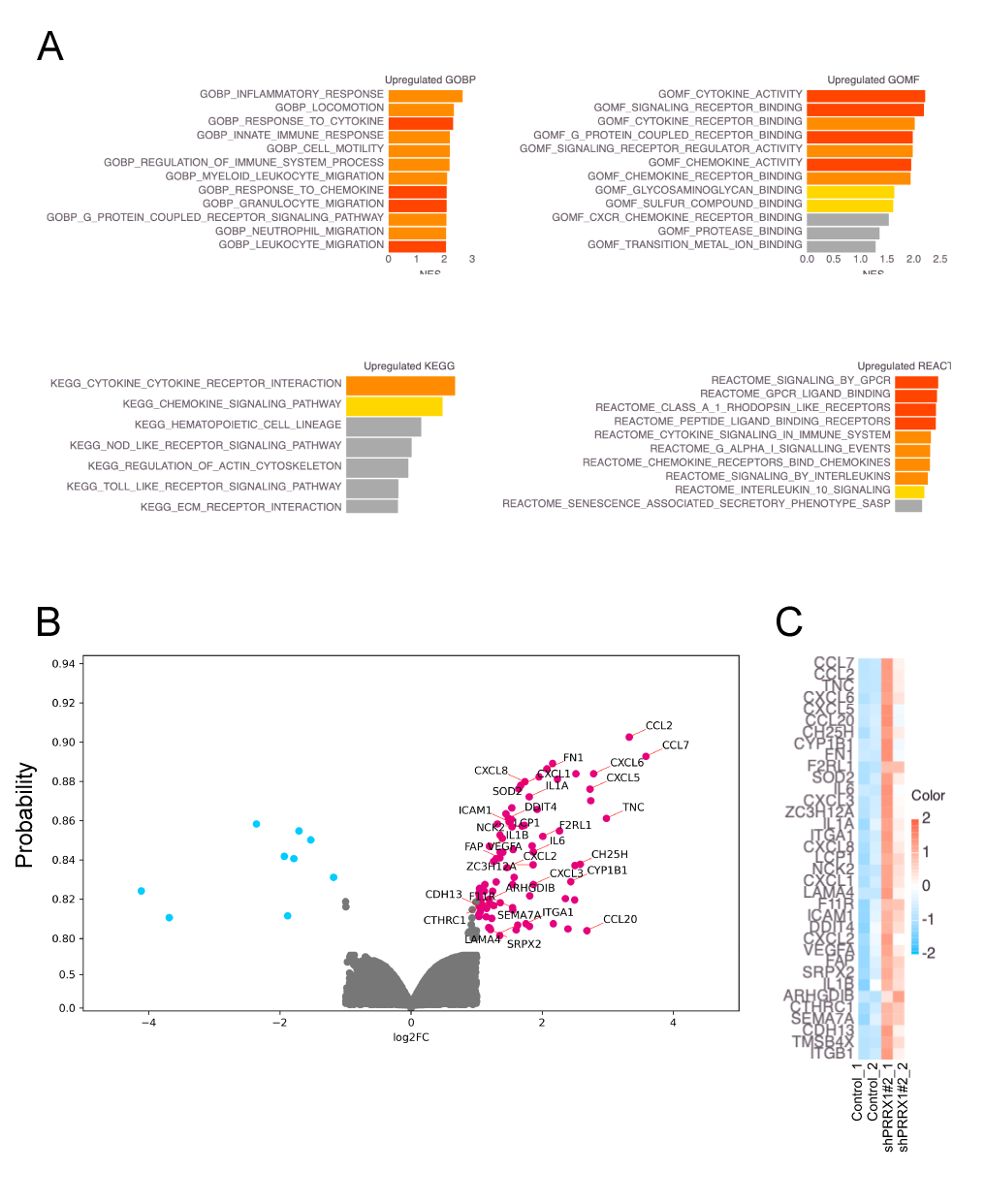


**Figure S3. RNA-seq analysis of PRRX1 Knockdown HS-PSS cells.**

(A) Gene Ontology (GO) enrichment analysis (Biological Process [GOBP], Molecular Function [GOMF]) and pathway analysis (Kyoto Encyclopedia of Genes and Genomes [KEGG], REACTOME) of downregulated genes between control vs PRRX1 knockdown samples are shown with bar plot. (B) Volcano plot of the differentially expression genes (DEGs) between control vs PRRX1 knockdown samples. The y-axis represents the posterior probability of DEGs and the fold change in log2 on the x-axis. Significantly downregulated genes (Probability > 0.8, logFC > 1) are in pink. (C) Heatmap of the DEGs related to the downregulated genes between control vs PRRX1 knockdown samples.


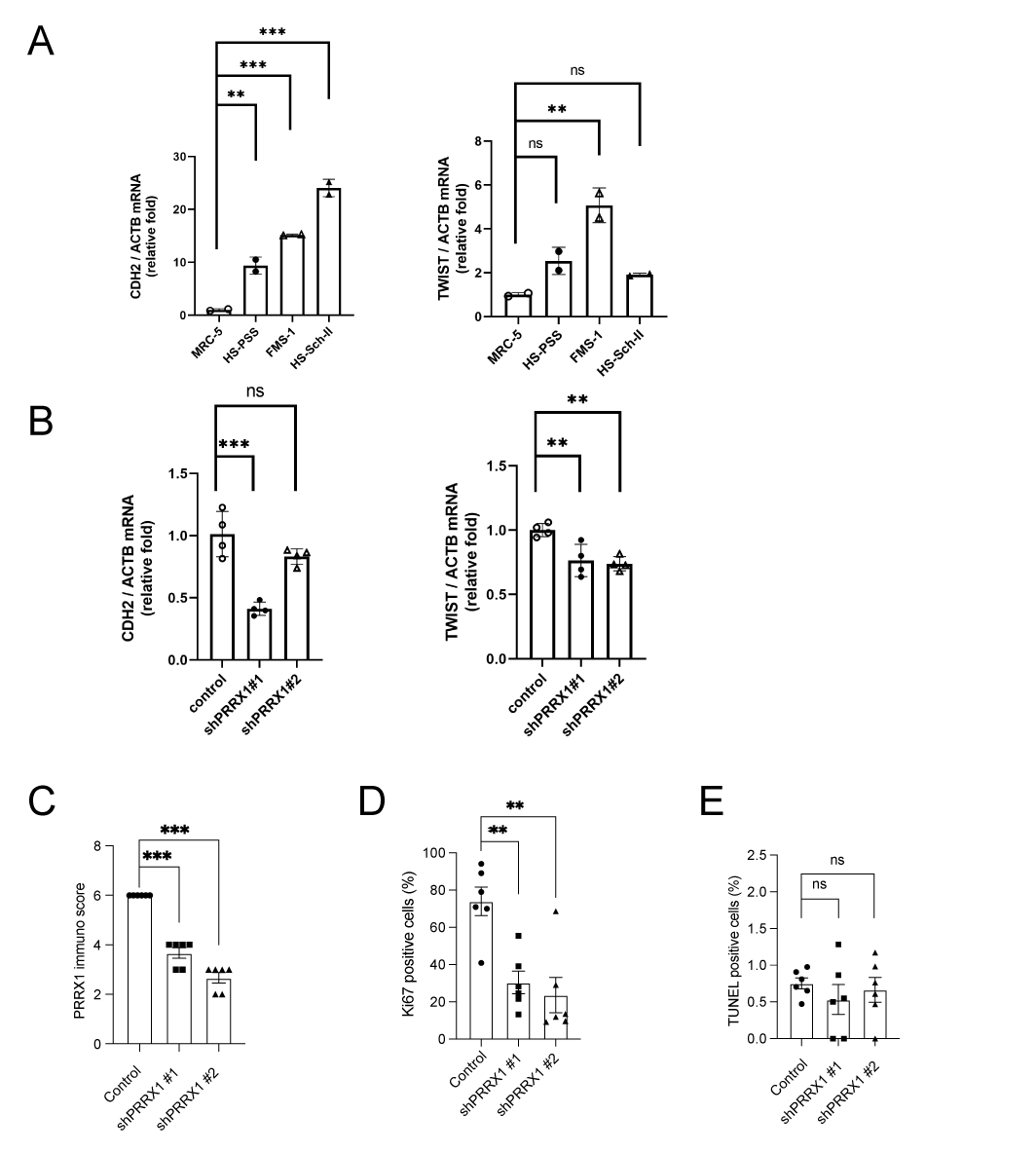


**Figure S4. Knockdown of PRRX1 suppresses mesenchymal properties and decreases tumorigenesis in a xenograft mouse model.**

(A, B) qPCR analysis of EMT-related genes (CDH2, TWIST) mRNA among each MPNST cell lines or knockdown cells. All values were normalized to ACTB mRNA levels (n = 4, two independent experiments). (C, D, E) Histological quantification of PRRX1 (immune score), Ki-67 or TUNEL (positive cells) in tumors developed from each cell line. Two or three fields in each tumor section were assessed (n = 6, three independent experiments). Data are presented as the means ± SEMs. ** p < 0.01; *** p < 0.001.


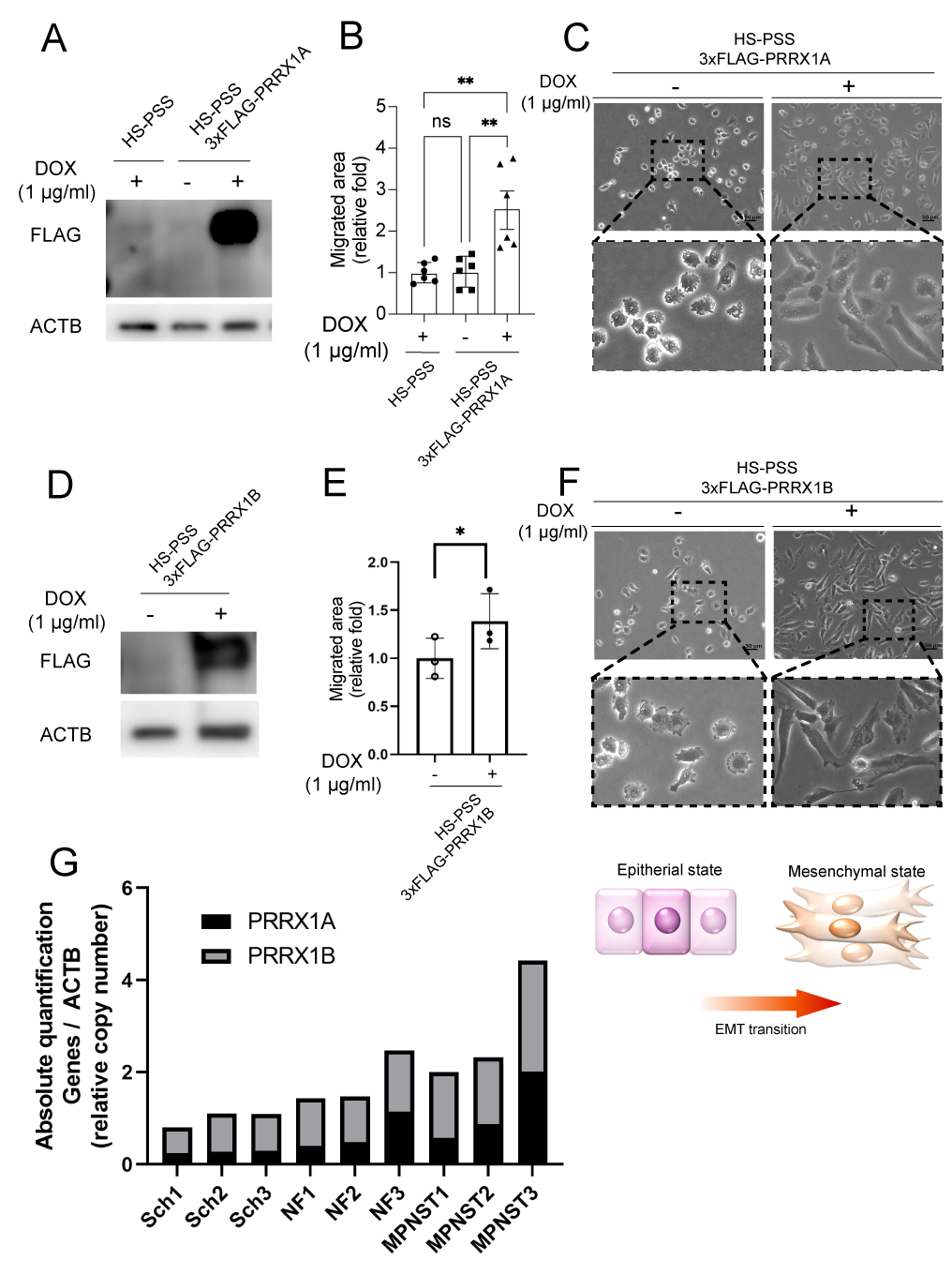


**Figure S5. PRRX1A and PRRX1B overexpression caused HS-PSS cells to increase their migration capacity and change to a mesenchymal-like cell shape.**

(A) Detection of 3xFLAG-PRRX1A by western blot analysis. HS-PSS/3xFLAG-PRRX1A cells were treated with 1 µg/mL doxycycline (DOX) for 1 day, and then total cell lysates were extracted to detect 3xFLAG-PRRX1A. (B) Comparison of migration capacity after 3xFLAG-PRRX1A overexpression by wound healing assay. After treating HS-PSS/3xFLAG-PRRX1A with 1 µg/mL DOX for 1 day, the assay was started. The relative migration of HS-PSS or HS-PSS/3xFLAG-PRRX1A (DOX(-)) was lower than that of HS-PSS/3xFLAG-PRRX1A (DOX(+)) at 24 h after scratch wound (n = 6, three independent experiments). (C) (Left) Assessment of cell morphological changes induced by PRRX1A overexpression. HS-PSS/3xFLAG-PRRX1A (DOX(+)) was changed to a mesenchymal cell-like appearance (Right). (C, D, E) The same experiment was performed on PRRX1B as PRRX1A. The schematic illustration shows the morphological changes of cells in the epithelial-mesenchymal transition. (G) qPCR analysis for the absolute quantification (relative copy number to ACTB, reference gene) of PRRX1A or PRRX1B in human schwannoma, neurofibroma, and malignant peripheral nerve sheath tumor (MPNST) tissues. Data are presented as the means ± SEMs. * p < 0.05; ** p < 0.01.


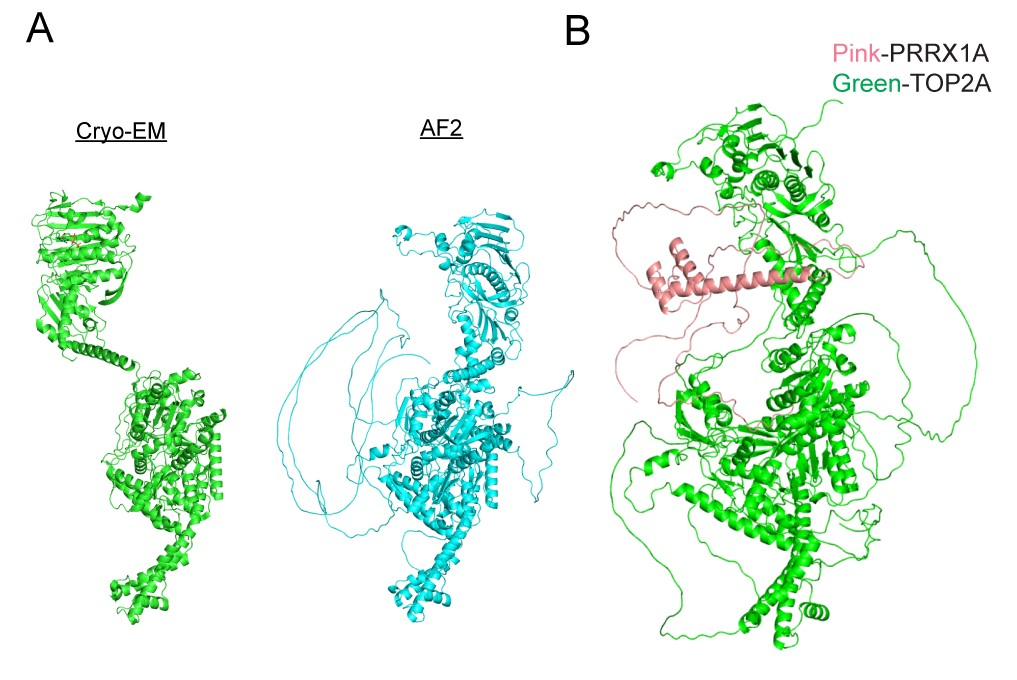


**Figure S6. Structural prediction by AF2 and FoldDock**

(A) Structural comparison of Cryo-EM and AF2. 6ZY8 was drawn as a monomer by PyMOL (green). One of the structures predicted by AF2 is shown (cyan). Both were merged and drawn to visualize the differences in structure. (B) Of the five PRRX1A-TOP2A complexes predicted by FoldDock, the most biologically relevant structure is shown. PRRX1A is shown in pink and TOP2A in green.


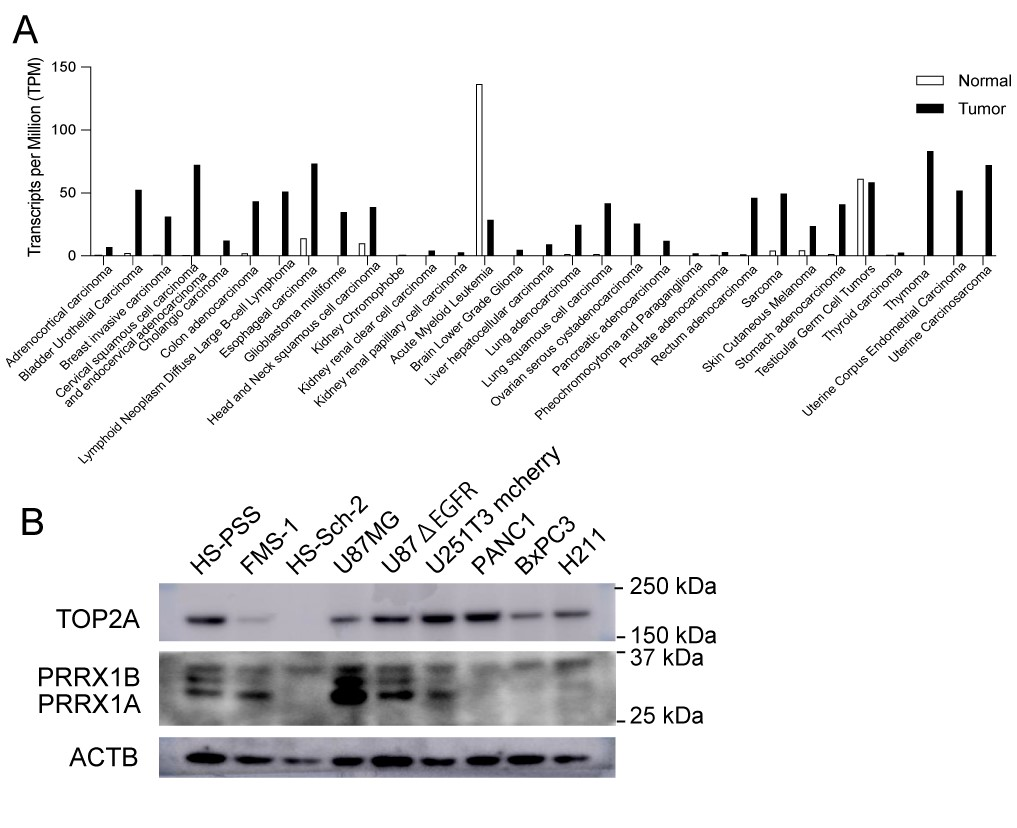


**Figure S7. TOP2A is a malignant factor in human MPNST**

(A) Expression profile of TOP2A mRNA in tumor samples. Data was obtained from the GEPIA Platform (http://gepia.cancer-pku.cn). (B) Western blot analysis of MPNST (HS-PSS, FMS-1, HS-Sch-2), glioblastoma (U87MG, U87 ΔEGFR, U251T3 mcherry), pancreatic cancer (PANC1, BxPC3), and small cell lung cancer (H211) cell lines. Whole cell lysates were extracted and the expression levels of PRRX1 and TOP2A, respectively, were compared.


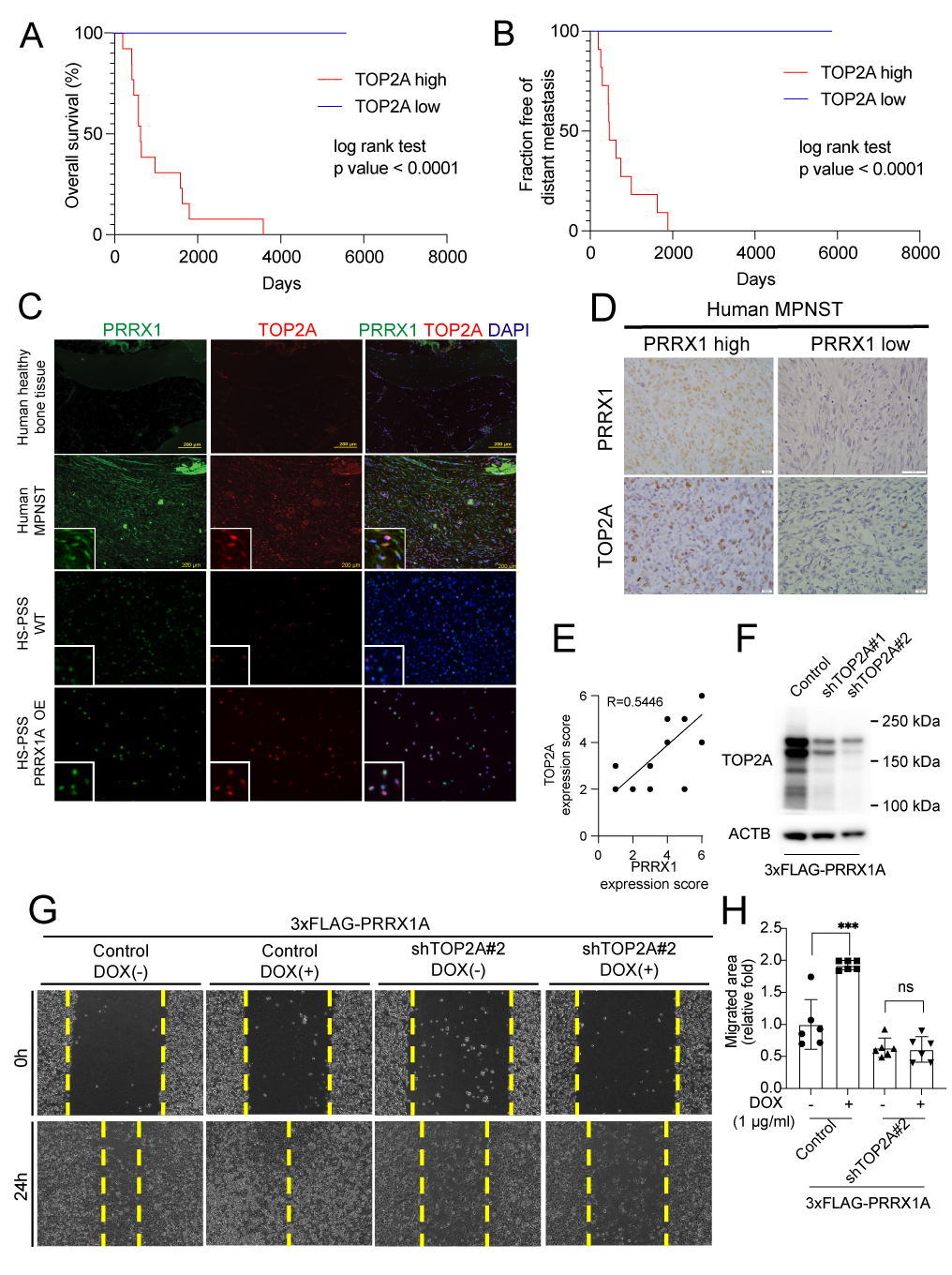


**Figure S8. The PRRX1–TOP2A interaction plays critical roles in migration and oncogenic signaling**

(A, B) Comparison of overall survival or distant metastasis between TOP2A-low and TOP2A-high MPNST patients. The Kaplan–Meyer survival curve demonstrates significant differences (log-rank test, P < 0.0001). (C) Dual fluorescence immunostaining for PRRX1 (green: AlexaFluor 488) and TOP2A (red: anti-mouse AlexaFluor 647) in human healthy bone, human MPNST tissues, HS-PSS/Wildtype and HS-PSS/3xFLAG-PRRX1A (DOX(+)). Enlarged image is at the bottom left. (D) Immunostaining of TOP2A in human MPNST tissues. Sections derived from human MPNSTs with high or low PRRX1 expression were stained with TOP2A, and representative images are shown. (E) Positive correlation of PRRX1 and TOP2A levels in human MPNST. To assess the correlation between PRRX1 and TOP2A, scores for the intensity of nuclear staining and the proportion of stained cells were added and compared. (F) Western blot analysis after TOP2A knockdown. HS-PSS/3xFLAG-PRRX1A cells were infected with lentivirus encoding each shTOP2A clone, and total cell lysates were extracted to compare the expression level of TOP2A. (G, H) Comparison of migration capacity after TOP2A knockdown by wound healing assay. HS-PSS/3xFLAG-PRRX1A/Control or HS-PSS/3xFLAG-PRRX1A/shTOP2A#2 cells were treated with 1 µg/mL doxycycline (DOX) for 1 day, and then the assay was started. (n = 6, three independent experiments). Data are presented as the means ± SEMs. *** p < 0.001.


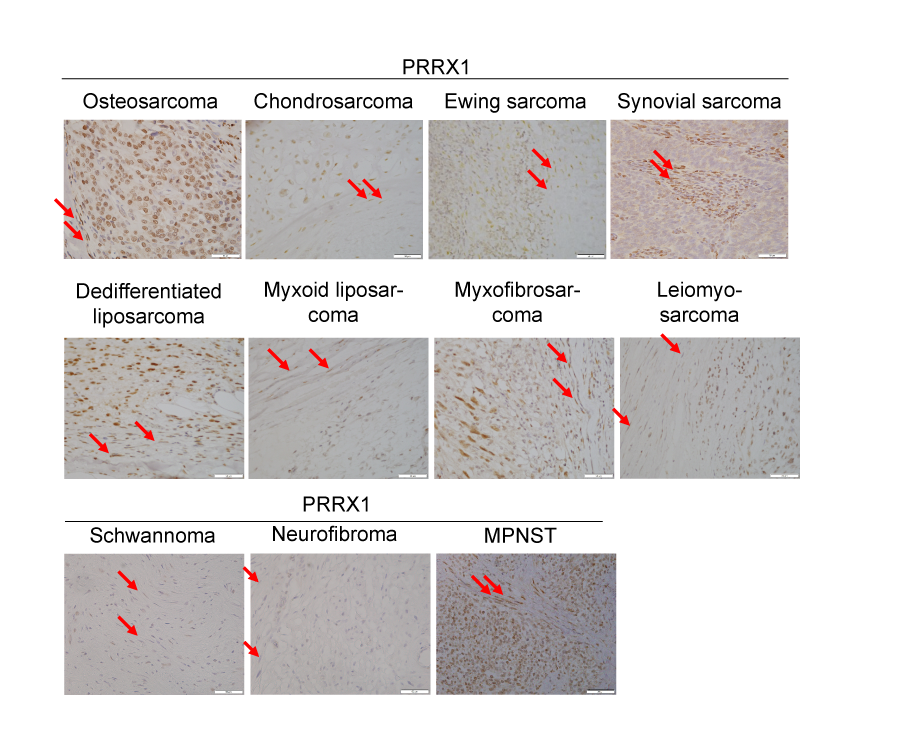


**Figure S9. PRRX1 immunostaining in the stroma of various tumor tissues.**

Sections derived from human sarcoma tissues were stained with PRRX1, and cancer-associated fibroblasts (CAFs) stained in the stroma were observed. Benign tumors like schwannoma and neurofibroma are poorly stained. Representative images are shown.


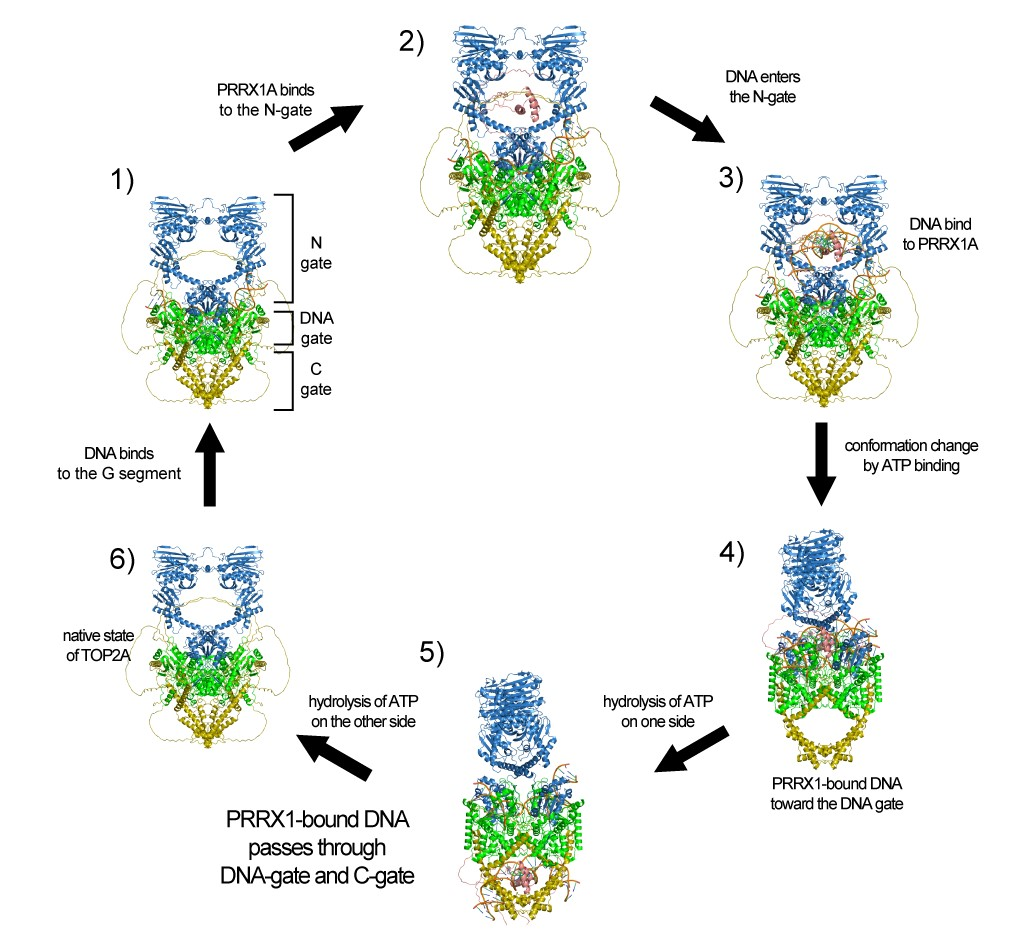


**Figure S10. Hypothesis for formation or release of the PRRX1–TOP2A interaction**

The molecular mechanism of the TOP2A and PRRX1A interaction is shown. The structure predicted by FoldDock and determined by cryo-EM were combined and drawn on PyMOL. PRRX1A is shown in pink. TOP2A is divided into three structures, with the N-gate highlighted in blue, the DNA-gate in green, and the C-gate in yellow. The process is as follows. 1) DNA binds to the G segment of TOP2A. 2) PRRX1A binds to the N-gate of TOP2A. 3) DNA enters the N-gate by association with PRRX1A. 4) The N-gate closes and rotates due to the binding of ATP; this physically pushes the PRRX1-bound DNA toward the DNA-gate. The linker between ATPase and TOPRIM physically blocks the N-gate and the DNA-gate, preventing PRRX1A-bound DNA from returning to the N-gate side. 5) PRRX1A-bound DNA passes through the DNA-gate after undergoing hydrolysis of ATP on one side. 6) ATP hydrolysis on the other side closes the DNA-gate and opens the C-gate, causing PRRX1A-bound DNA to leave TOP2A. ADP is removed from the ATPase and TOP2A returns to its native state. Return to 1).
